# In vitro peptidoglycan degradation assay using fluorescein-5-isothiocyanate-labelled sacculi

**DOI:** 10.1099/acmi.0.001178.v3

**Published:** 2026-07-15

**Authors:** Dema Alodaini, Adam Hart, Patrick J. Moynihan, Manuel Banzhaf

**Affiliations:** 1 School of Biosciences, University of Birmingham, Birmingham, UK; 2 Newcastle University Biosciences Institute, Newcastle University, Newcastle, UK

**Keywords:** peptidoglycan, peptidoglycan degradation, peptidoglycan hydrolases

## Abstract

We describe a method using fluorescein-5-isothiocyanate (FITC)-labelled peptidoglycan sacculi to characterize peptidoglycan hydrolases *in vitro*. In this protocol, soluble hydrolytic products released by peptidoglycan hydrolases are separated from the insoluble FITC-sacculi, quantified and set in relation to the overall available substrate. This enables the study of hydrolase activity in end-point or time-course experiments.

## Data Summary

 All data associated with this work are reported within the article.

## Introduction

Peptidoglycan degradation assays are frequently used to biochemically characterize peptidoglycan lytic enzymes. All assays have in common that they use peptidoglycan as a substrate but differ in their approach to detect the hydrolytic fragments. Hydrolytic peptidoglycan fragments can be separated by HPLC and quantified by mass spectrometry. This method is able to quantify the hydrolytic peptidoglycan fragments but lacks simplicity and throughput and is limited by the availability of HPLC instrumentation [1].

A simpler method to test peptidoglycan hydrolase activity is to use labelled sacculi. Most commonly, sacculi are either labelled with fluorescein-5-isothiocyanate (FITC) [2] or Remazol Brilliant Blue R [3] at free amines within peptidoglycan. Subsequently, soluble hydrolytic products released by peptidoglycan hydrolases can be measured spectrophotometrically after the insoluble substrate is removed by filtration or centrifugation. This enables the quantification of peptidoglycan fragment release, which in turn serves as a readout of hydrolase activity.

We purify our *Escherichia coli* peptidoglycan based on a protocol developed by Glauner, but other protocols that are more suitable for other organisms or experimental aims may be available [1]. Due to the ubiquity of amino groups in peptidoglycan, this method is adaptable to peptidoglycan from all bacteria. The protocols presented here are optimized for *E. coli* peptidoglycan. This method enables a high-throughput assay for detecting peptidoglycan hydrolase activity without requiring HPLC instrumentation; however, it does not permit detailed characterization of enzymatic specificity. In practice, the approach facilitates more rapid identification of hydrolase activity, which can subsequently be followed by targeted functional characterization using the method developed by Glauner.

## Materials

### *E. coli* peptidoglycan purification

1. **SDS solution**: 4% and 8% (w/v) dissolved in distilled water.
2. **PI buffer I**: 10 mM Tris-HCl, 10 mM NaCl, pH 7.0.
3. **Imidazole**: 3.2 M, pH 7.0
4. ***α*-Amylase**: 10 mg ml^−1^ in PI buffer I.
5. **Pronase E**: 10 mg ml^−1^ in 10 mM Tris-HCl, pH 7.2.
6. **Sodium azide (NaN_3_)**: 0.02% in distilled water.

### Peptidoglycan-FITC labelling

1. **Sodium bicarbonate buffer**: 0.5 M NaHCO_3_, pH 9–9.3. Dissolve 21 g of sodium bicarbonate and 0.36 g of sodium hydroxide in 400 ml distilled water. Adjust pH to 9 using NaOH and make up to a 500 ml final volume with distilled water.
2. **FITC dye**: powder. Store at −20 °C and protect from light, especially in solution.
3. **Purified PG**: see *E. coli* peptidoglycan purification.
4. **Ethanol**: 99.8%
5. **Acetone**: 99.8%

### Peptidoglycan degradation assay

1. **PG-FITC suspension**: final concentration of 10 mg ml^−1^ in sodium bicarbonate buffer (pH 9–9.3).
2. **PG reaction buffer**: 150 mM NaCl, 50 mM Tris-HCl, pH 8. Add 30 ml of distilled water into a sterile conical-bottom 50 ml Falcon tube. Weigh 0.39 g of NaCl and transfer to the Falcon tube. Weigh 0.27 g of Tris base and add it to the Falcon tube. Adjust pH to 8 and make up to a 45 ml final volume with distilled water. Filter (0.45 µm) and store at room temperature.
3. **pH retrieval buffer/sodium hydroxide**: 0.5 M NaOH, pH 9–9.3. Add 30 ml of distilled water into a sterile conical-bottom 50 ml Falcon tube. Weigh 0.8 g NaOH and transfer to the Falcon tube. Adjust pH using NaOH and make up to 40 ml final volume with distilled water. Filter and store at room temperature.
4. **Lysozyme from chicken egg white powder**: 10 mg ml^−1^ in PG reaction buffer.

## Methods

### *E. coli* peptidoglycan purification

1. Grow 3 l of exponential growth phase cells (OD_578_ 0.5/0.6) in a shaking incubator (37 °C, 180 r.p.m.).
2. Harvest cells by centrifugation (8,500 r.p.m., 4 °C, 20 min).
3. Resuspend the cell pellet in 9 ml of ice-cold distilled water.
4. Drop the cell suspension slowly into a 9 ml boiling 10% SDS solution and continue boiling for an additional 30 min to solubilize cell membranes and destroy high molecular weight DNA.
5. Allow the suspension to slowly cool to room temperature. However, avoid temperatures below 15 °C as SDS will precipitate and can no longer be removed by centrifugation (step 7).
6. Pellet the suspension to collect the PG sacculi by ultra-centrifugation (42,200 r.p.m., 40 min).
7. Remove the SDS by resuspending the pellet with distilled water and subsequently centrifuging the suspension (26,000 r.p.m., 4 °C, 45 min) to complete the wash step.
8. After the first wash, test the supernatant for the presence of SDS with the Hayashi test (*see* **Note 1**) and repeat step 7 until all SDS is removed.
9. Resuspend the pellet in 6.75 ml of PI buffer I.
10. Remove the glycogen by incubating cells with 750 µl of 3.2 M imidazole, pH 7.0 and 37.5 µl of 10 mg ml^−1^ *α*-amylase. Incubate at 37 °C for 30 min.
11. Remove the covalently linked lipoproteins by adding 50 µl of 10 mg ml^−1^ pronase E and incubating at 37 °C for 1 h (*see* **Note 2**).
12. Stop the reaction by adding 7.5 ml of 4% SDS and boiling the sample for 15 min.
13. Allow the suspension to cool to room temperature and wash off SDS as in step 7.
14. Resuspend the SDS-free pellet in a minimum of distilled water containing 0.02% NaN_3_ and store it at 4 °C or lyophilize. Lyophilized peptidoglycan can be stored at room temperature.

### Labelling sacculi with FITC fluorescent dye

1. In a round-bottom glass tube, mix purified PG with FITC powder using a weight ratio of 2 : 1.
2. Add 4 ml of 0.5 M sodium bicarbonate buffer to the PG-FITC mix.
3. Place a small magnetic stirrer inside the mix tube and incubate at 37 ˚C on a magnetic mixer for at least 4 h (*see* **Note 3**).
4. Wash unreacted FITC with sodium bicarbonate buffer by centrifugation (3,900 r.p.m., 4 °C, 15 min).
5. Discard supernatant and repeat step 4 until excess FITC is removed (*see* **Note 4**).
6. Wash twice using 99.8% ethanol as described in step 4.
7. Wash twice using 99.8% acetone as described in step 4.
8. Discard the acetone supernatant without disturbing the pellet and securely place the tube inside a biohazard hood to evaporate overnight.
9. Store the final product dry and away from light. Resuspend dried and labelled PG to the desired concentration in sodium bicarbonate buffer prior to use.

### Peptidoglycan degradation assay

1. Start by thawing the samples on ice (*see* **Note 5**).
2. In a conical-bottom 50 ml Falcon tube, prepare the PG master mix (*see* **Note 6**).
3. Add the desired protein concentration (1 μΜ is recommended) in a reaction tube, add 50 µl of the PG master mix and top up with PG reaction buffer to a final volume of 100 µl (*see* **Note 7**).
4. To prepare the positive control, add 8 µl lysozyme solution, 50 µl of the PG master mix and top up with PG reaction buffer to a final volume of 100 µl.
5. To prepare the negative control, add 50 µl of the PG master mix and top up with PG reaction buffer to a final volume of 100 µL.
6. Incubate all samples for 1 h in a heat block set to 37 °C at maximum speed (1500 r.p.m.), and cover with foil (*see* **Note 8**).
7. Transfer samples to a Millipore MultiScreen GV 96-well Filter Plate 0.22 µm. This filtration step separates the substrate from the enzymes to stop the reaction.
8. Place the filter plate on top of a 96-well Black Flat Bottom Microplate precisely (*see* **Note 9**).
9. Put the cover lid on the top of filter plate (*see* **Note 10**).
10. Centrifuge the plates for 3 min (2,500 r.p.m., at 4 °C) and use another set of plates for matching balance (*see* **Note 11**).
11. After centrifugation, samples are now filtered into the wells of the bottom black flat plate.
12. Gently add 50 µl of 0.5 M sodium hydroxide to each sample to normalize pH across all samples as FITC is pH sensitive (*see* **Note 12**).
13. Take the black flat plate containing the samples and measure emitted fluorescence using a plate reader (FITC fluorescence intensity is measured at 485 nm excitation and 525 nm emission).

## Notes

**Fig. 1.**
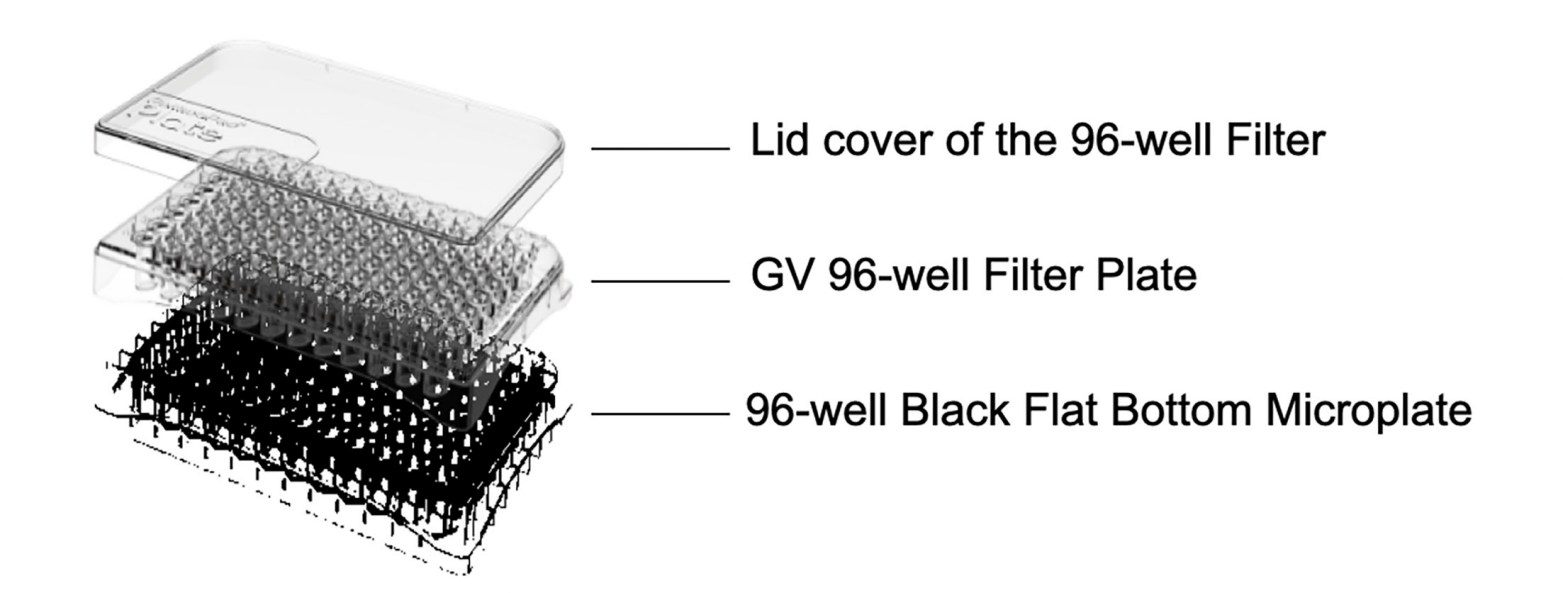
Schematic representation of the microtitre plate assembly used to ensure optimal centrifugation during the peptidoglycan degradation assay. From top to bottom, the assembly comprises a lid covering the GV 96-well filter plate, the GV 96-well filter plate, and a 96-well black flat-bottom microplate.

1. Hayashi test [4]: a sample of 335 µl was vigorously mixed with 170 µl of 0.7 M sodium phosphate, 7 µl of 0.5% methylene blue and 1 ml of chloroform. As the mixture settled, two phases could be observed. The sample was free of SDS when the bottom organic phase (chloroform) showed a slight pink colour. In the presence of SDS, the organic phase showed a blue colour.
2. Pronase E was pre-incubated at 60 °C for 2 h to eliminate possible contaminants.
3. Wrap the tube with foil or incubate in the dark. All FITC-containing reagents should be shielded from light.
4. Continue washing with water until residual FITC is no longer detected/the wash is clear and not orange.
5. Work on ice during the whole assay.
6. Master mix can be calculated as [5 µl FITC-PG suspension (10 mg ml^−1^) + 45 µl PG reaction buffer] × no. of samples.
7. Make sure to vortex the PG-FITC master mix very well before its addition to the protein sample.
8. The incubation time can be varied depending on the hydrolase activity of the tested enzymes.
9. Precise stacking of filter and black plates during centrifugation is important for samples to be filtered correctly into the wells of the black plate and not end up between the wells.
10. Assemble plates as in Fig. 1.
11. The balance plate must include both filter and black flat plates, along with the lid cover to match the weight of the test plate.
12. Change tips from one well to another and make sure to mix after adding the NaOH, without creating bubbles.

## Abbreviations

FITC: fluorescein-5-Isothiocyanate
PG: peptidoglycan
